# Mobile Health Daily Life Monitoring for Parkinson Disease: Development and Validation of Ecological Momentary Assessments

**DOI:** 10.2196/15628

**Published:** 2020-05-11

**Authors:** Jeroen Habets, Margot Heijmans, Christian Herff, Claudia Simons, Albert FG Leentjens, Yasin Temel, Mark Kuijf, Pieter Kubben

**Affiliations:** 1 Department of Neurosurgery School of Mental Health and Neuroscience Maastricht University Maastricht Netherlands; 2 Department of Psychiatry and Neuropsychology School for Mental Health and Neuroscience Maastricht University Maastricht Netherlands; 3 GGzE Institute for Mental Health Care Eindhoven Eindhoven Netherlands; 4 Department of Neurology Maastricht University Medical Center Maastricht Netherlands; 5 Department of Neurosurgery Radboud University Medical Center Nijmegen Netherlands

**Keywords:** ecological momentary assessment, experience sampling method, electronic diary, Parkinson’s disease monitoring

## Abstract

**Background:**

Parkinson disease monitoring is currently transitioning from periodic clinical assessments to continuous daily life monitoring in free-living conditions. Traditional Parkinson disease monitoring methods lack intraday fluctuation detection. Electronic diaries (eDiaries) hold the potential to collect subjective experiences on the severity and burden of motor and nonmotor symptoms in free-living conditions.

**Objective:**

This study aimed to develop a Parkinson disease–specific eDiary based on ecological momentary assessments (EMAs) and to explore its validation.

**Methods:**

An observational cohort of 20 patients with Parkinson disease used the smartphone-based EMA eDiary for 14 consecutive days without adjusting free-living routines. The eDiary app presented an identical questionnaire consisting of questions regarding affect, context, motor and nonmotor symptoms, and motor performance 7 times daily at semirandomized moments. In addition, patients were asked to complete a morning and an evening questionnaire.

**Results:**

Mean affect correlated moderate-to-strong and moderate with motor performance (R=0.38 to 0.75; *P*<.001) and motor symptom (R=0.34 to 0.50; *P*<.001) items, respectively. The motor performance showed a weak-to-moderate negative correlation with motor symptoms (R=−0.31 to −0.48; *P*<.001). Mean group answers given for on-medication conditions vs wearing-off-medication conditions differed significantly (*P*<.05); however, not enough questionnaires were completed for the wearing-off-medication condition to reproduce these findings on individual levels.

**Conclusions:**

We presented a Parkinson disease–specific EMA eDiary. Correlations between given answers support the internal validity of the eDiary and underline EMA’s potential in free-living Parkinson disease monitoring. Careful patient selection and EMA design adjustment to this targeted population and their fluctuations are necessary to generate robust proof of EMA validation in future work. Combining clinical Parkinson disease knowledge with practical EMA experience is inevitable to design and perform studies, which will lead to the successful integration of eDiaries in free-living Parkinson disease monitoring.

## Introduction

### Background

Parkinson disease is a neurodegenerative disorder that is characterized by bradykinesia, rigidity, and tremor. Many patients develop fluctuations in cardinal motor symptoms, such as bradykinesia, tremor, and postural instability, and levodopa-induced dyskinesia [1,2]. Nonmotor symptoms may also show fluctuations during the day [3,4]. Current gold standards in symptom monitoring, such as the Movement Disorders Society (MDS)–Unified Parkinson Disease Rating Scale and the Parkinson Disease Quality of Life-39, are suboptimal to detect such fluctuations over short periods, as they cover a longer temporal domain and require active observed tasks [5,6]. Monitoring methods that can also detect motor and nonmotor fluctuations over shorter periods in free-living conditions can contribute to applying personalized medicine in Parkinson disease [7,8]. Examples of such new methods are telemonitoring [9] and mobile health (mHealth) apps, often including wearable sensors [10-12]. Patients with neurological conditions are believed to be able to use mobile apps [13]; however, the quality, validation, and usability of the available apps are often low [14]. Nonetheless, there have been promising results of using mHealth monitoring systems for motor and nonmotor symptoms of Parkinson disease during free-living situations [15-17].

Electronic diaries (eDiaries) hold the potential to contribute to these new monitoring methods by collecting valuable information on motor symptoms [18,19] and nonmotor symptoms in free-living conditions [16].

Recently published recommendations on Parkinson disease eDiary development by a specific MDS Task Force and Committee underline the relevance and potential of this approach [20,21]. Ecological momentary assessment (EMA), also referred to as an experience sampling method, is a method that collects subjective experiences at multiple, semirandomized moments during a day. Commonly used in psychiatric and psychological populations, it holds the potential for somatic diseases as well [22]. The scarce literature describing EMA in Parkinson disease reports feasibility in small cohorts of up to 5 patients. Reproduction and further investigation of the usefulness and value of EMA in Parkinson disease are needed [4,16,23].

### Objective

We developed the first specific Parkinson disease eDiary using EMA and set the first steps to validate the EMA method in a broad Parkinson disease cohort.

## Methods

### Study Population

We included 20 patients who were diagnosed with Parkinson disease following the UK Parkinson disease Society Brain Bank Diagnostic Criteria, who were aged between 18 and 80 years, who possessed a smartphone (at least Android 4 or iPhone operating system 8), and who had adequate proficiency in the Dutch language. A Montreal Cognitive Assessment scale score lower than 24 was the only exclusion criterion [24]. Demographic and general disease characteristics were collected, such as sex and age, Parkinson disease duration, levodopa equivalent daily dosage (LEDD), number of daily dopaminergic medication intake moments, presence of intraday motor fluctuations, and recent Hoehn and Yahr scores.

### Ecological Momentary Assessment Study Design

Participants enrolled between August 2018 and March 2019 and participated for 14 consecutive days. EMA questionnaires (referred to as beeps) were presented at seven semirandomized moments a day, one beep within every block of 2 hours between 8 AM and 10 PM. The questionnaire had to be opened within 15 min after notification to prevent procrastination. A separate morning questionnaire was available between 4 AM and 1 PM, and an evening questionnaire was available between 8 PM and 4 AM. Answers on statement questions were given on a 7-point Likert scale. The EMA method was executed via the smartphone app, PsyMate [25]. EMA was combined with the use of three wearable sensors containing accelerometers and gyroscopes. Technical details of the protocol design and feasibility analyses are reported earlier [26]. The study protocol was conducted following the Helsinki guidelines and was approved by the local medical ethical committee of Maastricht University Medical Center+.

### Parkinson Disease–Specific Ecological Momentary Assessment Questionnaire

To the best of our knowledge, no specific EMA questionnaire for Parkinson disease exists. On the basis of a literature search and structured interviews with clinical experts, patients, and caregivers, both focused on parameters that differentiate *good* vs *bad* Parkinson moments, we determined the content of the Parkinson disease EMA questionnaire. We consulted an EMA expert group to phrase the specific questions and design the EMA method.

### Data Preparation

Patients with a completion rate lower than 33% were excluded from analyses [27]. Beeps containing missing values because of unfinished questionnaires or digital data transmission failure were excluded.

To analyze positive and negative affect, we calculated the mean of the items *feeling well*, *feeling cheerful*, and *feeling relaxed* and the mean of the items *feeling down*, *feeling fearful*, and *feeling stressed*, respectively. To analyze general motor function, we calculated the mean of the items *ability to perform current activity*, *ability to walk well*, *ability to talk well*, and *to experience steady mobility*. When we refer to the items *mean positive affect*, *mean negative affect*, or *general motor function* in the paper, we are referring to these calculated mean scores. General motor function in the evening questionnaire was calculated as the mean of the evening questionnaire items *ability to dress*, *ability to eat*, *ability to do household activities*, *ability to do personal care*, and *ability to walk*. The evening questionnaire items *experienced many off periods* and *experienced long off periods* were averaged in an item representing off-moment severity during the day.

To represent the change in an item since the last beep, we calculated differences over time scores. The answer to the previous beep (t−1) was subtracted from the answer of the current beep (t). Two beeps are, on average, 2 hours separated from each other. We did not calculate the difference in scores between the first completed beep a day and the last beep of the previous day.

### Statistical Analysis

We analyzed means, standard deviations, and distributions per item. A skewed distribution of answers of an item to the minimum (1) or the maximum (7) is called a floor or a ceiling effect, respectively. If present, we evaluated whether this floor or ceiling effect could be expected and could be accepted or might be based on an invalid, nonspecific, or nonsensitive question and deserved further evaluation.

To validate whether items measure what they are intended to measure, the correlation between an item and a gold standard that measures the same concept can be assessed. If this expected correlation is present, this means the construct validity of that item is proven [20,28]. As there are no validated assessment scales that assess Parkinson disease symptoms as frequent as our EMA beeps, there is a lack of a gold standard measure. Therefore, we assessed the construct validity by analyzing correlations between items from the same beep that are expected to correlate based on clinical knowledge. To further analyze construct validity, we analyzed correlations between the mean answer over all beeps during 1 day and the answer from the corresponding evening questionnaire. For the latter, we excluded days without the completed evening questionnaire. As the theoretically expected correlation of sleep with other symptoms is ambiguous, we excluded the morning questionnaires from validation analyses.

We compared beep answers given in different medication conditions to explore differences in symptom severity. We merged the two transition conditions, from on-medication to off-medication and vice versa, to differentiate 3 conditions: on-medication condition, off-medication condition, and the transition between on- and off-medication condition.

By calculating correlations between scores of items that are expected to correlate, we analyzed the sensitivity of our EMA questionnaire to measure changes over time. We explored the differences between beep answers given in different medication conditions, on-medication condition, off-medication condition, and transitions between the two. The beeps identified as off-medication condition represent the wearing-off-medication condition because the patients were never fully depleted of dopaminergic medication. In the rest of the paper, we will use the term on-beeps and off-beeps to refer to these medication conditions during a completed beep questionnaire. We performed these comparisons on group and individual levels. The significance of differences between the different medication conditions was calculated using Mann-Whitney U tests. Correlations were calculated using Spearman correlation tests. *P* values were corrected with a Bonferroni correction. All the data preparation and statistical analyses were performed in Python Jupyter Notebook 3 using packages pandas (version 0.24.2), Numpy (version 1.16.4), datetime (version 1.0.0), and Scipy (version 1.3.0).

## Results

### Study Population

We included 4 female and 16 male patients with idiopathic Parkinson disease with a mean age of 63 years (SD 7), a mean disease duration of 8 years (SD 6), and a mean LEDD of 770 mg (SD 394); 6 participants were treated with deep brain stimulation for a mean period of 3.3 years (SD 1.5; Table 1). The mean completion rate was 78% out of 98 continuous beeps (SD 12). No participants were excluded based on a too low completion rate (ie, completion rate <33%) [27].

**Table 1:** Demographics of study population.

Demographics		Values
Gender ratio (female:male)		4:16
Age (years), mean (SD)		63 (7)
Disease duration (years), mean (SD)		8 (6)
Levodopa equivalent daily dosage (mg), mean (SD)		770 (394)
**Deep brain stimulation**		
	Patients with deep brain stimulation treatment, n	6
	Duration of deep brain stimulation treatment (years), mean (SD)	3.3 (1.5)
**Hoehn and Yahr scale, n (%)**		
	1	2 (10)
	1.5	2 (10)
	2	7 (35)
	2.5	3 (10)
	3	3 (15)
	4	1 (5)
Montreal Cognitive Assessment, mean (SD)		27.6 (1.5)

### Parkinson Disease Ecological Momentary Assessment Questionnaire Development

Affect and context items from widely applied EMA questionnaires in psychiatry were added [29]. Parkinson disease–specific items are based on a literature search and structured interviews with clinicians, patients, and caregivers. A detailed description of this literature search and the structured interviews can be found in Multimedia Appendix 1.

Repeated discussions with the *EMA expert group* in our institution (among them CS) gave us the following insights into designing a valid EMA questionnaire for patients with Parkinson disease: (1) do not only assess motor symptoms by direct questions about the specific motor symptom, (2) include assessment of the burden or the influence of the symptoms on the patient’s performance/well-being, and (3) include items on context (where/with whom/what) and affect and to have the possibility to correct for varying settings or mood fluctuations. On the basis of the advice of the EMA expert group, we consistently phrased the questions as statements in the “I” perspective and tried to avoid confirming (“I do feel...”) and denying (“I do not feel...”) statements next to each other [27,30]. Furthermore, when translating clinical terms or items from retrospective questionnaires into EMA items, we aimed to maximize face validity by using everyday language. The final EMA questionnaire is shown in Textbox 1.

**Parkinson disease ecological momentary assessment questionnaire content, English translation from the original Dutch version. The beep questionnaire, which is presented seven times during the day, represents the four motor domains as well as affect, cognition, context, and motor performance. The evening questionnaire covers off-moments and motor performance over the day, and the morning questionnaire covers sleep.**

**Beep questionnaire (semi-random repeated moments)**

- I feel well
- I feel down
- I feel fearful
- I feel stressed
- I feel sleepy
- I am tired
- I am cheerful
- I am relaxed
- I can concentrate well
- I experience hallucinations
- I am at [home, work, travelling, at family/friend’s place, in public]
- I am with [nobody, family, partner, colleagues, friends]
- I am doing [work, resting, household/odd jobs, sports, something else]
- I can do this without hinder
- I am comfortable walking/standing
- I can sit or stand still easily
- I can speak easily
- I can walk easily
- I experience tremor
- I am moving slow
- I experience stiffness
- My muscles are tensioned
- I am uncontrollable moving
- I feel … [1: OFF, 2: ON -> OFF, 3: ON, 4: OFF -> ON]
- I took Parkinson medication since last beep [yes, no, I don’t recall]

**Morning questionnaire**

- I slept well
- I woke up often last night
- I feel rested
- It was physically difficult to get up
- It was mentally difficult to get up

**Evening questionnaire**

- I had long OFF periods today
- I had many OFF periods today
- Walking went well today
- (un)dressing went well today
- Eating/ drinking went well today
- Personal care went well today
- Household activities went well today
- I was tired today

### Parkinson Disease Ecological Momentary Assessment Questionnaire Validity

**Figure 1:**
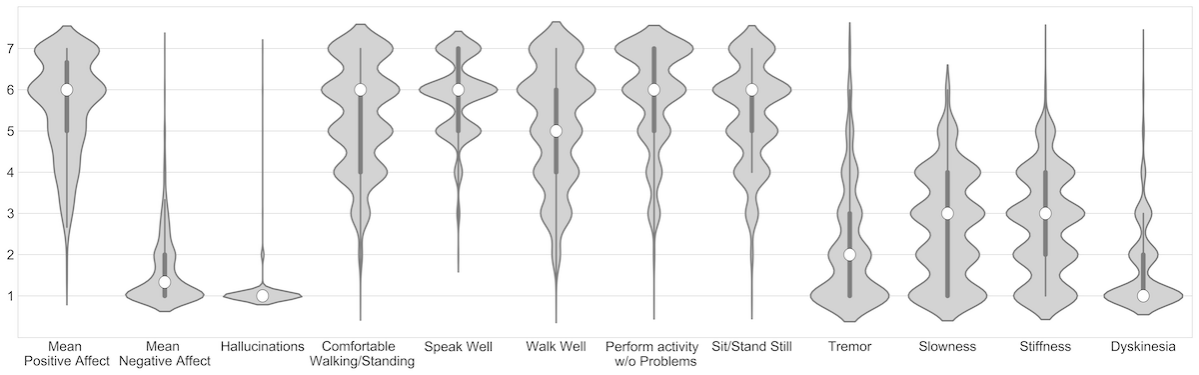
Distribution plots of answers from beep questionnaires. Mean positive and negative affect showed high and low mean answers, respectively. The ability to perform daily life tasks showed moderate-to-high mean answers, whereas the motor symptom items showed low-to-moderate mean answers. All items were statements and were answered on a 7-point Likert scale, ranging from 1 (not at all) to 7 (very). The white dot represents the median answer, the thick black line represents the IQR, and the thin black lines represent the rest of the distribution, calculated as IQR times 1.5. The width of the shapes correlates with the probability that the patient answered the corresponding value.

Figure 1 shows means and distributions of all participants per item from the beep questionnaire and the evening questionnaire. Positive affect shows a small ceiling effect, whereas negative affect shows a floor effect. Experiencing hallucinations shows a strong floor effect, with only one participant experiencing hallucinations. Positive formulated items on motor functioning show a small floor effect. Experiencing tremor and dyskinesia shows stronger floor effects than experiencing slowness and stiffness.

**Figure 2:**
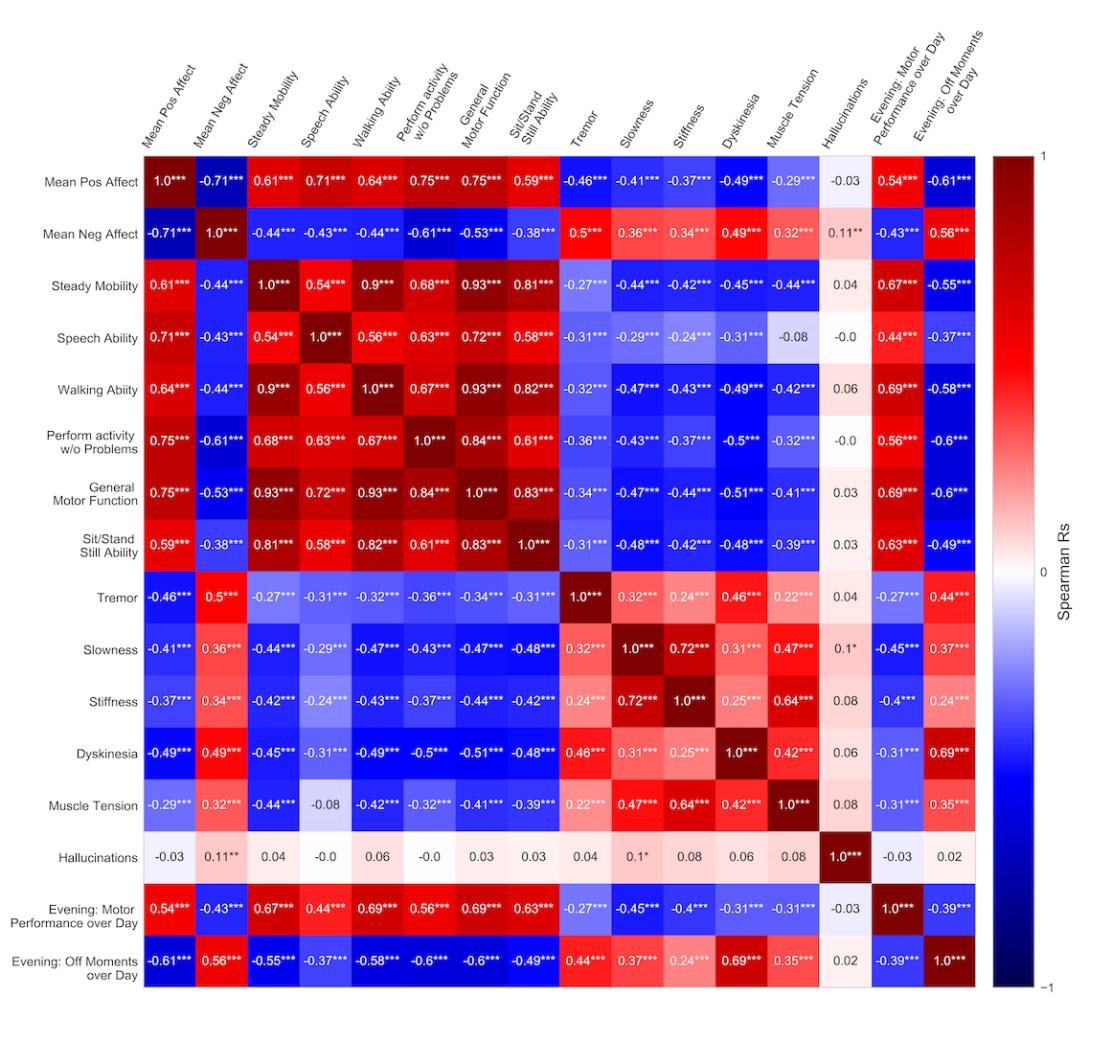
Correlations between items from the beep questionnaire and the evening questionnaires. We observed strong and moderate correlations between the motor performance items and mean positive and negative affect, respectively. We observed correlations between mean affect scores, motor symptoms, motor performance, and medication states, in the beep questionnaires and the evening questionnaires, in directions that were expected.

Construct validity was assessed by evaluating the presence of expected correlations between items (Figure 2). Mean positive and negative affect showed a strong negative correlation with each other (R=−0.71; *P*<.001; Figure 2). Both positive and negative affect scores showed moderate-to-strong correlations with general motor functioning (R=0.75 and R=−0.53, respectively; *P*<.001). Mean positive and negative affect scores showed weak-to-moderate correlations with different motor symptoms (R=−0.37 to −0.49 and R=0.32 to 0.50, respectively; *P*<.001). General motor functioning showed moderate-to-weak correlations with the motor symptoms tremor, slowness, stiffness, and dyskinesia (R=−0.34, −0.47, −0.44, and −0.51, respectively; *P*<.001).

Beep answers on mean affect scores and general motor functioning from 1 day showed moderate correlations with both the items assessing the amount of experienced off-beeps and the general motor performance from the corresponding evening questionnaires in expected directions (R=−0.43 to 0.69; *P*<.001). Beep answers during the day on slowness, stiffness, tremor, and dyskinesia showed weak-to-moderate correlations with general motor functioning answers from the evening questionnaire (R=−0.24 to 0.44; *P*<.001). These items assessing motor symptoms in the beep questionnaires also showed weak-to-moderate correlations in the expected directions with the item assessing off-beeps in the evening questionnaire (R=0.24 to 0.69; *P*<.001). Although dyskinesia is not a typical symptom during off-beeps, it correlated strongly with off-beeps over the whole day (R=0.69; *P<*.001).

The correlations between difference over time scores were less strong as the absolute answers (see Multimedia Appendix 1 for a correlation heatmap of difference over time scores). All correlations were weak to absent.

### Influence of Medication Condition on Ecological Momentary Assessment Answers

**Figure 3:**
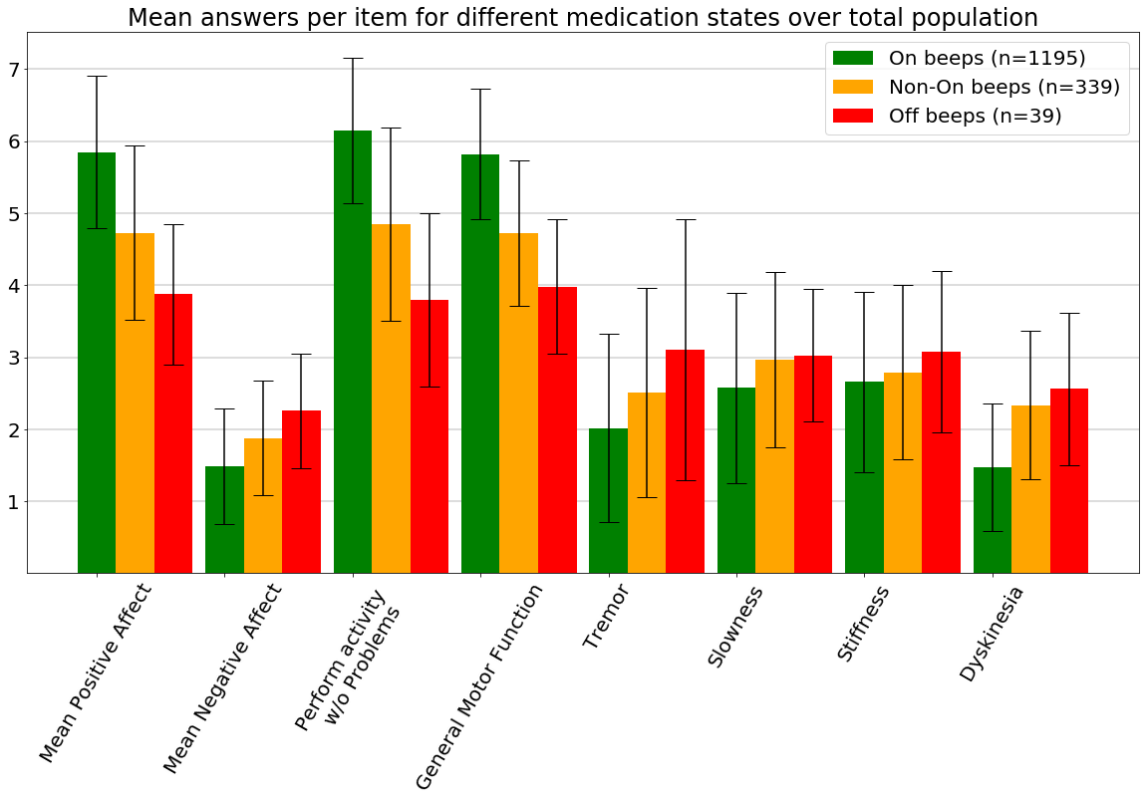
Mean answers during different medication states. The given answers in different medication conditions show significant differences. The direction of differences is as expected, except for dyskinesia, which is scored higher, on average, during off-condition compared with on-condition. Whiskers indicate standard deviations. On-beeps represent answers during on-medication, non–on-beeps represent answers during off-medication and during the transition phase, and off-beeps represent answers only during off-medication.

Of 1573 beeps, 1195 (75.97%) were labeled by patients as *answered in on-medication condition* (on-beeps), 339 (21.56%) were labeled by patients as *answered in between on- and off-medication condition* (transition beeps), and 39 (2.48%) were labeled by patients as *answered in off-medication condition* (off-beeps; Figure 3). On a group level, mean answers significantly differed between on-beeps and non–on-beeps for mean positive affect, general motor function, slowness, and dyskinesia. Mean answers between on-beeps and off-beeps significantly differed for mean positive affect, mean negative affect, general motor function, and tremor.

On an individual level, mean answers during different medication conditions did not differ significantly. The differences were either not significant or not relevant. Only 5 of 20 participants reported 20% or more beeps in the *non–on-medication condition* (Multimedia Appendix 1).

## Discussion

### Clinical Relevance of Ecological Momentary Assessment for Free-Living Parkinson Disease Monitoring

Owing to the fluctuating nature of Parkinson disease and its heterogeneous character, EMA holds theoretically great potential to increase insight into symptom severity and burden fluctuation during free-living conditions. Our work fits in the first milestone defined by the MDS Technology Task Force and the MDS Rating Scales Program Electronic Development Ad-Hoc Committee by giving insight into the prioritization of outcomes, which are relevant for the patient to measure [20]. Obviously, this paper is one of the first of many to follow.

A common challenge for all future work in this field is the validation of methods and questionnaires. Validated scales only exist for Parkinson disease monitoring with longer time intervals than the short time intervals needed to detect intraday fluctuations. This fact makes classical validation with golden standards difficult and even incorrect depending on the methodology. The MDS Task Force on Technology, therefore, advices to validate new Parkinson disease monitoring methods for free-living conditions according to accuracy, reliability, sensitivity, and minimal clinically significant differences [21]. This validation challenge is also relevant for the integration of additional biometric monitor devices, such as accelerometers, gyroscopes, microphones, or electrophysiological monitor devices. Vizcarra et al [20] make a distinction between the integration of action-dependent and action-independent monitoring. It is expected that creating golden standards for action-dependent tasks in, for example, a laboratory setting is easier than creating standards for an action-independent setting such as free-living [18,19]. For the latter, validated Parkinson disease monitoring devices collecting subjective experiences on symptom severity and burden can be of substantial value.

The most applied and promising action-independent Parkinson disease monitoring methods for free-living conditions are based on wearable sensors [31,32]. Attempts to include subjective diary data in the validation of sensor data algorithms were hindered by practical limitations mainly, for example, recall bias and diary fatigue [19,33]. Smartphone-based EMA methodology can be applied less obtrusively and address these traditional diary limitations. Naturally, the feasibility of this method is heavily dependent on the frequency and duration in which it is applied. These factors require thorough future investigations and may differ per intention of use, for example, wearable sensor calibration or periodic free-living monitoring of nonmotor symptoms.

### General Lessons on Ecological Momentary Assessment in Parkinson Disease

The introduction of a new method in Parkinson disease monitoring entails challenges and questions beyond the current literature. To address these challenges and questions as effectively as possible, we gathered a multidisciplinary team consisting of those with clinical Parkinson disease expertise (experts in neurology, neuropsychiatry, and neurosurgery and specialized nurses) and experienced practitioners of EMA (neuropsychology and neuropsychiatry). We described our most important lessons regarding the content and the phrasing of the EMA questionnaire to inform clinicians and researchers interested in applying EMA in Parkinson disease. Moreover, a recently published checklist provides researchers with a tool to design an EMA-based diary study [34]. Essential for EMA in Parkinson disease is the similarity between the frequency of EMA assessments and the frequency of symptom fluctuations that are intended to capture. Thus, EMA studies may require different designs depending on whether they monitor levodopa-induced dyskinesia fluctuations over a day or whether they monitor the effect of an extra levodopa agonist on morning bradykinesia.

### Validation of Ecological Momentary Assessment in Parkinson Disease

Mean answer values and distributions show expected findings (Figure 1). Positive affect items are known to be answered higher than negative affect items [35]. The high mean answers on general motor function and the low mean answers on motor symptoms can be explained by the stable treated population and the relatively low overall disease progression (Table 1). Concerning the observed floor and ceiling effects, we only regard the item on hallucinations as obsolete for this population because of the observed extreme floor effect. As stated earlier, negative affect items are known to show a floor effect. Tremor and dyskinesia also show an unsatisfying floor effect, although we think this is because of the low prevalence of these symptoms in our sample. Moreover, the unexpected positive correlation between dyskinesia and experienced off-beeps suggests that the dyskinesia item might not be well understood by patients. Limited awareness on the presence of dyskinesia among patients with Parkinson disease is described earlier [36]. This finding might also be strengthened by the low prevalence of dyskinesia in the population. We advise, therefore, to avoid the use of nonapplicable, general questions for individual patients. If an item is not applicable for a patient, the patient should be clearly instructed on how to answer that item.

The moderate-to-high correlations present between affect, motor function, and motor symptoms prove the construct validity of the Parkinson disease EMA method partially. The low-to-moderate correlations between motor function and motor symptoms warrant cautious conclusions, and follow-up validation among a narrower selected population with more motor fluctuations is needed to more extensively proof construct validity.

The high number of beeps answered in on-medication condition (Figure 3) and the weak till absent correlations between difference over time scores (Multimedia Appendix 1) confirm this hypothesis. Significance levels are calculated using Mann-Whitney *U* tests (all *P*<0.5). All questions except *Stiffness* differed significantly between on-beeps and non–on-beeps. All questions except *Slowness* and *Stiffness* differed significantly between on-beeps and off-beeps. Ideally, the significant differences that were only found on the group level also hold on individual levels in the next validation study, especially because EMA is intended for individual monitoring.

Despite the fact that further investigation is needed, EMA in Parkinson disease seems to be potentially useful and valid when evaluating the moderate-to-high correlations between affect, general functioning, bradykinesia, and stiffness. Altogether, we interpret our findings as encouraging, and we stress the importance of a careful patient selection depending on the exact goal of EMA monitoring.

### Limitations

The broad inclusion policy was a well-considered choice in the study design, and it resulted in important information about the feasibility and validity of EMA in a broad Parkinson disease population. When applied in a more specified cohort, clinimetric validation analyses necessary for the next step in validation are better feasible, such as principal component analyses to exclude fewer sensitive items. The latter may lead to individual patient- or patient subgroup–specific questionnaire content.

### Conclusions

EMA-based eDiaries are promising to enrich free-living Parkinson disease monitoring with essential information on motor and nonmotor fluctuations. First validation analyses suggest the internal validation of EMA among a general Parkinson disease population. Careful patient selection and EMA design adjustment to this targeted population and their fluctuations are necessary to generate robust proof of EMA validation in future work. Combining clinical Parkinson disease knowledge with practical EMA experience is inevitable to design and perform studies, which will lead to the successful integration of eDiaries in free-living Parkinson disease monitoring.

## Appendix

Multimedia Appendix 1 Supplementary material.

## Abbreviations

eDiary: electronic diary
EMA: ecological momentary assessment
LEDD: levodopa equivalent daily dosage
MDS: Movement Disorders Society

## References

[1] Jankovic J (2005). Motor fluctuations and dyskinesias in Parkinson's disease: clinical manifestations. Mov Disord.

[2] Metman LV (2002). Recognition and treatment of response fluctuations in Parkinson's disease: review article. Amino Acids.

[3] Kim A, Kim H, Shin CW, Kim A, Kim Y, Jang M, Jung YJ, Lee W, Park H, Jeon B (2018). Emergence of non-motor fluctuations with reference to motor fluctuations in Parkinson's disease. Parkinsonism Relat Disord.

[4] van der Velden RM, Broen MP, Kuijf ML, Leentjens AF (2018). Frequency of mood and anxiety fluctuations in Parkinson's disease patients with motor fluctuations: A systematic review. Mov Disord.

[5] Goetz CG, Tilley BC, Shaftman SR, Stebbins GT, Fahn S, Martinez-Martin P, Poewe W, Sampaio C, Stern MB, Dodel R, Dubois B, Holloway R, Jankovic J, Kulisevsky J, Lang AE, Lees A, Leurgans S, LeWitt PA, Nyenhuis D, Olanow CW, Rascol O, Schrag A, Teresi JA, van Hilten JJ, LaPelle N, Movement Disorder Society UPDRS Revision Task Force (2008). Movement Disorder Society-sponsored revision of the Unified Parkinson's Disease Rating Scale (MDS-UPDRS): scale presentation and clinimetric testing results. Mov Disord.

[6] Hagell P, Nygren C (2007). The 39 item Parkinson's disease questionnaire (PDQ-39) revisited: implications for evidence based medicine. J Neurol Neurosurg Psychiatry.

[7] Sánchez-Ferro Á, Elshehabi M, Godinho C, Salkovic D, Hobert MA, Domingos J, van Uem JM, Ferreira JJ, Maetzler W (2016). New methods for the assessment of Parkinson's disease (2005 to 2015): A systematic review. Mov Disord.

[8] Dorsey ER, Vlaanderen FP, Engelen LJ, Kieburtz K, Zhu W, Biglan KM, Faber MJ, Bloem BR (2016). Moving Parkinson care to the home. Mov Disord.

[9] Schneider RB, Biglan KM (2017). The promise of telemedicine for chronic neurological disorders: the example of Parkinson's disease. Lancet Neurol.

[10] Cancela J, Mascato SV, Gatsios D, Rigas G, Marcante A, Gentile G, Biundo R, Giglio M, Chondrogiorgi M, Vilzmann R, Konitsiotis S, Antonini A, Arredondo MT, Fotiadis DI (2016). Monitoring of motor and non-motor symptoms of Parkinson's disease through a mHealth platform. Conf Proc IEEE Eng Med Biol Soc.

[11] Tsiouris KM, Gatsios D, Rigas G, Miljkovic D, Seljak BK, Bohanec M, Arredondo MT, Antonini A, Konitsiotis S, Koutsouris DD, Fotiadis DI (2017). PD_Manager: an mHealth platform for Parkinson's disease patient management. Healthc Technol Lett.

[12] Ferreira JJ, Godinho C, Santos AT, Domingos J, Abreu D, Lobo R, Gonçalves N, Barra M, Larsen F, Fagerbakke Ø, Akeren I, Wangen H, Serrano JA, Weber P, Thoms A, Meckler S, Sollinger S, van Uem J, Hobert MA, Maier KS, Matthew H, Isaacs T, Duffen J, Graessner H, Maetzler W (2015). Quantitative home-based assessment of Parkinson's symptoms: the SENSE-PARK feasibility and usability study. BMC Neurol.

[13] Winberg C, Kylberg M, Pettersson C, Harnett T, Hedvall P, Mattsson T, Lexell EM (2017). The use of apps for health in persons with multiple sclerosis, Parkinson's disease and stroke - barriers and facilitators. Stud Health Technol Inform.

[14] Rey ML, Vela-Desojo L, Cuerda RC (2019). Mobile phone applications in Parkinson's disease: A systematic review. Neurologia.

[15] Zhan A, Mohan S, Tarolli C, Schneider RB, Adams JL, Sharma S, Elson MJ, Spear KL, Glidden AM, Little MA, Terzis A, Dorsey ER, Saria S (2018). Using smartphones and machine learning to quantify Parkinson disease severity: the mobile Parkinson disease score. JAMA Neurol.

[16] Broen MP, Marsman VA, Kuijf ML, van Oostenbrugge RJ, van Os J, Leentjens AF (2016). Unraveling the relationship between motor symptoms, affective states and contextual factors in Parkinson's disease: a feasibility study of the experience sampling method. PLoS One.

[17] Lipsmeier F, Taylor KI, Kilchenmann T, Wolf D, Scotland A, Schjodt-Eriksen J, Cheng W, Fernandez-Garcia I, Siebourg-Polster J, Jin L, Soto J, Verselis L, Boess F, Koller M, Grundman M, Monsch AU, Postuma RB, Ghosh A, Kremer T, Czech C, Gossens C, Lindemann M (2018). Evaluation of smartphone-based testing to generate exploratory outcome measures in a phase 1 Parkinson's disease clinical trial. Mov Disord.

[18] Rodríguez-Molinero A, Pérez-López C, Samà A, de Mingo E, Rodríguez-Martín D, Hernández-Vara J, Bayés À, Moral A, Álvarez R, Pérez-Martínez DA, Català A (2018). A kinematic sensor and algorithm to detect motor fluctuations in Parkinson disease: validation study under real conditions of use. JMIR Rehabil Assist Technol.

[19] Del Din S, Godfrey A, Mazzà C, Lord S, Rochester L (2016). Free-living monitoring of Parkinson's disease: Lessons from the field. Mov Disord.

[20] Vizcarra JA, Sánchez-Ferro Á, Maetzler W, Marsili L, Zavala L, Lang AE, Martinez-Martin P, Mestre TA, Reilmann R, Hausdorff JM, Dorsey ER, Paul SS, Dexheimer JW, Wissel BD, Fuller RL, Bonato P, Tan AH, Bloem BR, Kopil C, Daeschler M, Bataille L, Kleiner G, Cedarbaum JM, Klucken J, Merola A, Goetz CG, Stebbins GT, Espay AJ, MDS Technology Task Forcethe MDS Rating Scales Program Electronic Development Ad-Hoc Committee (2019). The Parkinson's disease e-diary: Developing a clinical and research tool for the digital age. Mov Disord.

[21] Espay AJ, Hausdorff JM, Sánchez-Ferro Á, Klucken J, Merola A, Bonato P, Paul SS, Horak FB, Vizcarra JA, Mestre TA, Reilmann R, Nieuwboer A, Dorsey ER, Rochester L, Bloem BR, Maetzler W, Movement Disorder Society Task Force on Technology (2019). A roadmap for implementation of patient-centered digital outcome measures in Parkinson's disease obtained using mobile health technologies. Mov Disord.

[22] Verhagen SJ, Hasmi L, Drukker M, van Os J, Delespaul PA (2016). Use of the experience sampling method in the context of clinical trials. Evid Based Ment Health.

[23] van der Velden RM, Mulders AE, Drukker M, Kuijf ML, Leentjens AF (2018). Network analysis of symptoms in a Parkinson patient using experience sampling data: An n = 1 study. Mov Disord.

[24] Nasreddine ZS, Phillips NA, Bédirian V, Charbonneau S, Whitehead V, Collin I, Cummings JL, Chertkow H (2005). The Montreal Cognitive Assessment, MoCA: a brief screening tool for mild cognitive impairment. J Am Geriatr Soc.

[25] PsyMate [NL].

[26] Heijmans M, Habets JG, Herff C, Aarts J, Stevens A, Kuijf ML, Kubben PL (2019). Monitoring Parkinson's disease symptoms during daily life: a feasibility study. NPJ Parkinsons Dis.

[27] Palmier-Claus JE, Myin-Germeys I, Barkus E, Bentley L, Udachina A, Delespaul PA, Lewis SW, Dunn G (2011). Experience sampling research in individuals with mental illness: reflections and guidance. Acta Psychiatr Scand.

[28] Ginty A, Gellman MD, Turner JR (2013). Construct validity. Encyclopedia of Behavioral Medicine.

[29] Simons CJ, Hartmann JA, Kramer I, Menne-Lothmann C, Höhn P, van Bemmel AL, Myin-Germeys I, Delespaul P, van Os J, Wichers M (2015). Effects of momentary self-monitoring on empowerment in a randomized controlled trial in patients with depression. Eur Psychiatry.

[30] Delespaul AE (1995). Assessing Schizophrenia in Daily Life: The Experience Sampling Method.

[31] Ramdhani RA, Khojandi A, Shylo O, Kopell BH (2018). Optimizing clinical assessments in Parkinson's disease through the use of wearable sensors and data driven modeling. Front Comput Neurosci.

[32] Thorp JE, Adamczyk PG, Ploeg H, Pickett KA (2018). Monitoring motor symptoms during activities of daily living in individuals with Parkinson's disease. Front Neurol.

[33] Papapetropoulos SS (2012). Patient diaries as a clinical endpoint in Parkinson's disease clinical trials. CNS Neurosci Ther.

[34] Janssens KA, Bos EH, Rosmalen JG, Wichers MC, Riese H (2018). A qualitative approach to guide choices for designing a diary study. BMC Med Res Methodol.

[35] Jacobs N, Menne-Lothmann C, Derom C, Thiery E, van Os J, Wichers M (2013). Deconstructing the familiality of variability in momentary negative and positive affect. Acta Psychiatr Scand.

[36] Hoff JI, van Hilten BJ, Roos RA (1999). A review of the assessment of dyskinesias. Mov Disord.

