# Supplementary material for: Mobile Health Daily Life Monitoring for Parkinson Disease: Development and Validation of Ecological Momentary Assessments
Source: JMIR Mhealth Uhealth. 2020 May 11;8(5):e15628. doi: 10.2196/15628 (PMC7248801; doi:10.2196/15628)
Supplement: Multimedia Appendix 1 [file mhealth_v8i5e15628_app1.docx]

**Supplemental material PD EMA validation paper**

**1. Development of Parkinson’s disease (PD) specific Ecological Momentary Assessment (EMA) list**

To determine the content of a PD EMA questionnaire we performed a literature search, structured interviews with clinical experts, patients and caregivers and consulted the EMA expert group within our institution.

We searched the Pubmed database in February 2018 with the following search strategy: ‘*(("Ecological Momentary Assessment"[Mesh]) OR ((experience sampling method) OR (ESM) OR (EMA) OR (ecological momentary assessment))) AND (("Parkinson Disease"[Mesh]) OR ("Movement Disorders"[Mesh]) OR parkinson* OR (movement disorder*))*’. This resulted in 47 hits, of which one described EMA in PD.^1^ Even this study did not use a specialized EMA questionnaire for PD. Next, we reviewed literature describing relevant aspects for PD monitoring at home. The SENSE-PARK research group published two well-conducted studies on relevant parameters in PD monitoring at home. To define what characterizes ‘good’ and ‘bad’ Parkinson moments at home, they performed a web-survey among 198 patients followed by 6 focus groups, and performed a Delphi-study among 12 clinicians. They extracted six domains to monitor: gait, bradykinesia, tremor, sleep, sway, cognition.^2^ To define which parameters and assessment tools are needed to monitor these six domains, they performed a 2-round Delphi-study among 12 clinicians, 159 patients and 72 caregivers.^3^

Ferreira et al asked PD patients, caregivers and clinicians to score the importance of parameters in PD monitoring in the home situation between 1 and 5, and they asked them to rate a top 3 most important parameters. Here, we present a list of parameters that are scored higher than 4.0 on average and are mentioned in the top 3 parameters in more than 33% of the subjects. They are presented in the second column of table 1.

We performed twenty structured interviews with individual patients and asked them ‘*What complaint distinguishes a good from a bad Parkinson moment the most?*’ and ‘*What restriction that limits you in daily life distinguishes a good from a bad Parkinson moment the most?*’. We performed six structured interviews with movement disorders experts within our academic hospital, and asked their opinion about the same questions. Results on how many percent of patients and clinical experts mentioned symptoms to monitor are shown in table 1.

|  | **SENSE-PARK research group** | | **Own structured interviews** | |
| --- | --- | --- | --- | --- |
| **Parameter** | Serrano et al^2^* | Ferreira et al^3^** | % of patients | % of experts |
| stiffness/ slowness | 1.7/ 2.2 |  | 80 | 83 |
| walking |  | 4.1, 43% | 45 | 100 |
| Freezing of gait |  | 4.3, 59% |  |  |
| dyskinesia | 0.1 |  | 40 | 67 |
| tremor | 1.5 | 4.1, 36% | 40 | 100 |
| tired | 1.5 |  | 35 | 33 |
| executive functioning | 0.6 | 4.7, 98% | 25 | 33 |
| clothing/washing |  | 4.7, 91% | 25 | 83 |
| housekeeping |  | 4.7, 91% | 25 | 50 |
| balance/falls | 1.0 | 4.6, 81% | 20 | 83 |
| fine motor movements | 1.7 | 4.5, 55% | 20 | 33 |
| speech | 0.5 |  | 15 | 83 |
| pain (legs) | 1.0 |  | 15 | 17 |
| activities |  | 4.7, 91% | 10 | 83 |
| writing |  | 3.9, 43% | 5 | 0 |
| autonomous | 1.9 |  | 5 | 17 |
| ON vs OFF |  |  | 0 | 50 |
| Sleep |  | 4.4, 66% | 0 | 0 |

**Table 1: Overview of results of different authors on domains and parameters of importance in PD monitoring in the home environment**

*: Serrano et al present their results as a combined score between the open survey round and the focus group results.

**: Ferreira et al present their results as the average score (between 1 and 5) and the percentage the parameter was rated in the top 3 parameters to monitor.

As described in the manuscript, we distilled specific questions about (motor) performance and (motor) symptoms based on the results shown in table 1.

**2. Original Dutch EMA questionnaire**

**Figure 1: Original PD specific EMA list in Dutch**

**3. Additional figures of statistical analyses**

**Sensitivity to change analyses with difference over time scores**

**
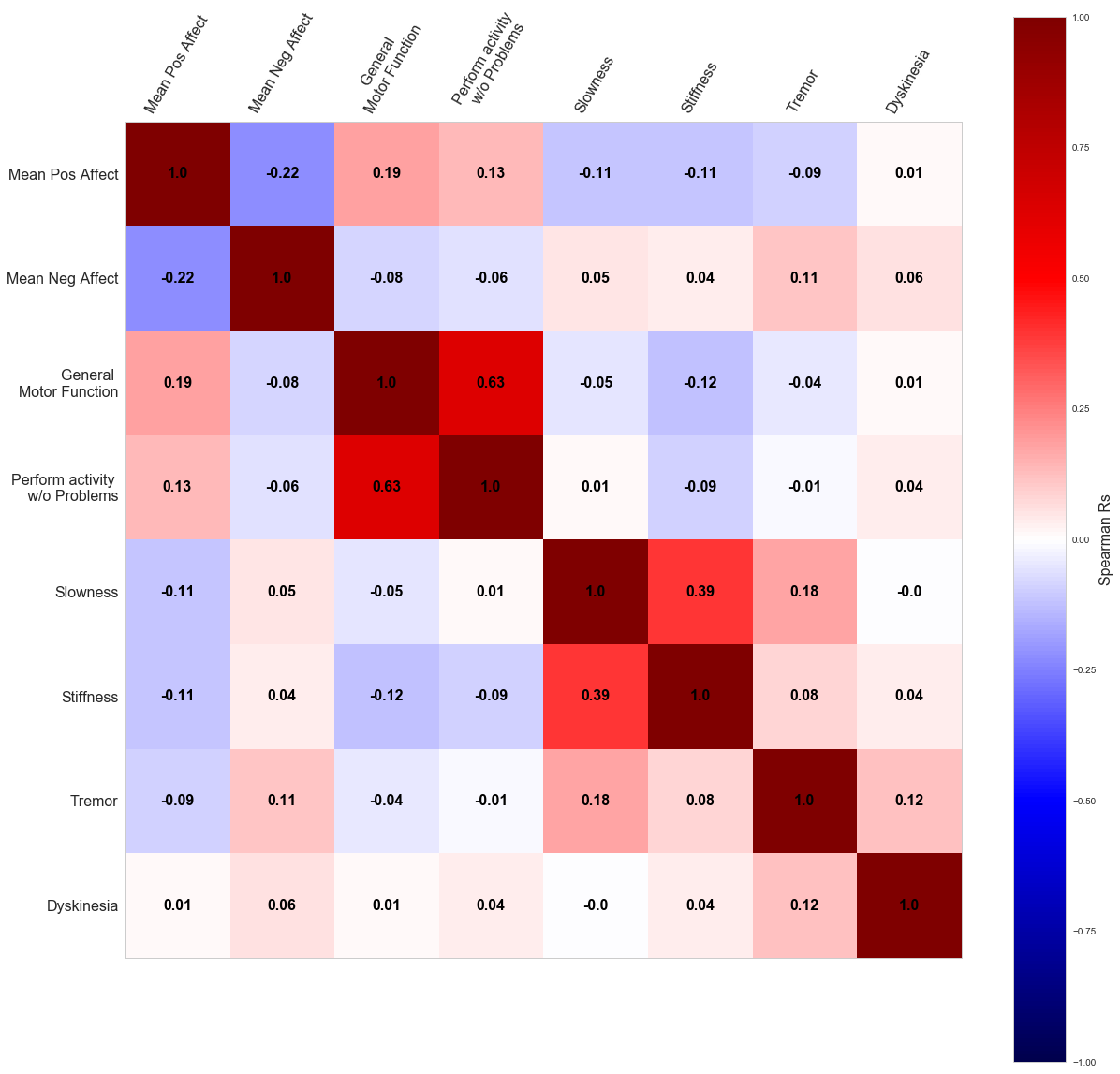
**

**Figure 2: Correlations between difference over time scores**

Changes in mean positive affect were weakly correlated with changes in mean negative affect (R = -0.22). Mean positive and negative affect changed parallel with the motor symptoms in the expected directions, although the correlations were very weak till weak (R = 0.01 – 0.22). Changes since the previous beep in general function only correlated weakly with mean positive affect (R = 0.19) and were not correlated with the rest of the items. Changes in slowness and stiffness did correlate with each other (R = 0.39) and correlated weakly with changes in tremor (R = 0.18 and 0.08).


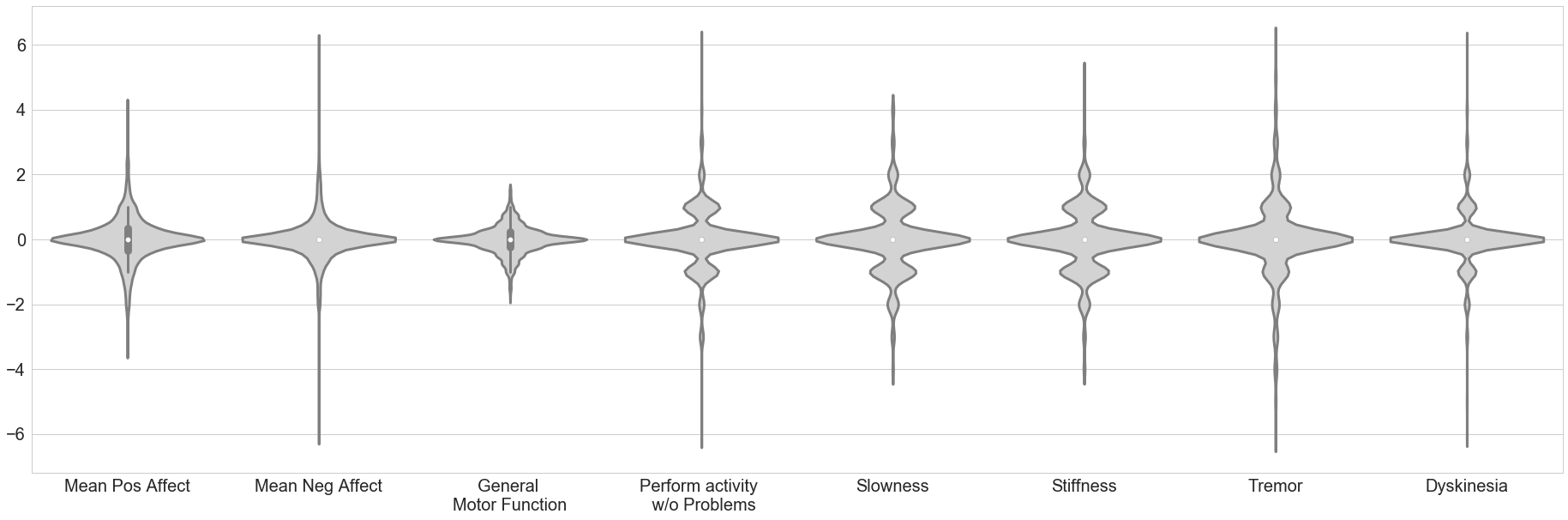


**Figure 3: Distribution plot for difference over time scores of most relevant items.** The very high relative amount of (nearly) 0 answers means that the answers on these items did not show large differences compared to the answers on the same items of the prior completed questionnaire. The percentage of beeps with a difference over time score of ‘0’ was 45% for mean positive affect, 55% for mean negative affect, 41% for general motor functioning, 57% for slowness, 56% for stiffness, 65% for tremor and 76% for dyskinesia.

**4. Detailed analysis of answers given during different medication conditions**


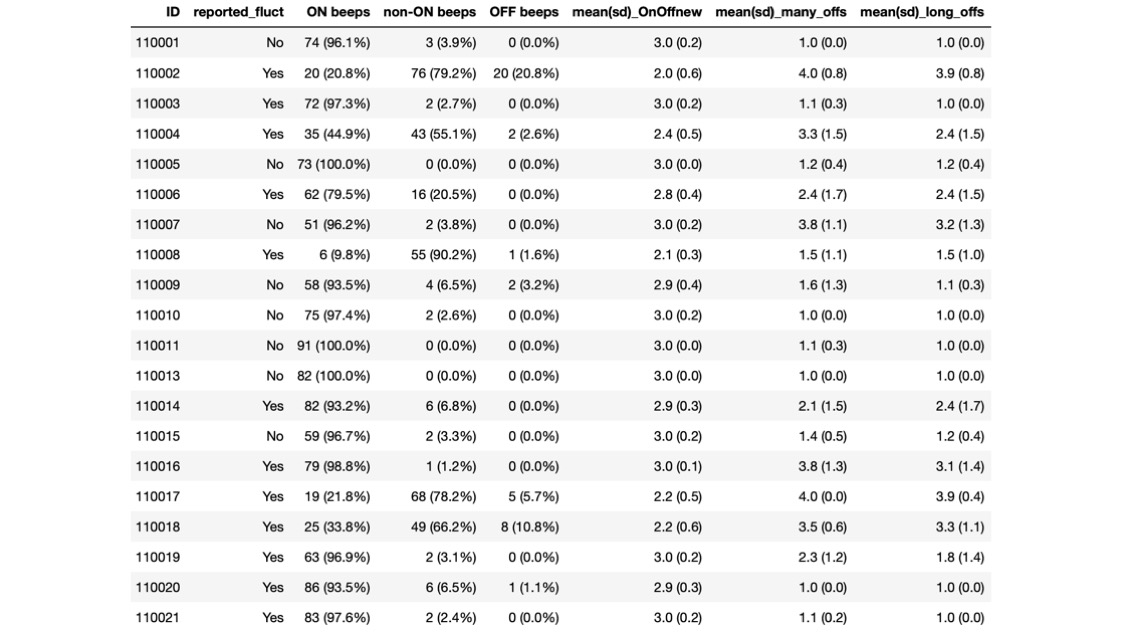


**Figure 4: Subjective and objective presence of ON/OFF motor fluctuations.** The percentage of non-ON beeps and OFF-beeps is insufficient to make valid comparisons on an individual level in most of the participants. Furthermore, there are large differences between the reported ON- and OFF-states and the experienced OFF-moments over days between patients who self-reported to have fluctuations. This prohibits On- vs Off-state analyses on individual levels and only allows us to make an analysis on group level, which cannot be used to draw hard conclusions.

**References**

1. Broen MP, Marsman VA, Kuijf ML, Van Oostenbrugge RJ, van Os J, Leentjens AF. Unraveling the Relationship between Motor Symptoms, Affective States and Contextual Factors in Parkinson's Disease: A Feasibility Study of the Experience Sampling Method. PloS one 2016;11(3):e0151195.

2. Serrano JA, Larsen F, Isaacs T, et al. Participatory design in Parkinson's research with focus on the symptomatic domains to be measured. Journal of Parkinson's disease 2015;5(1):187-196.

3. Ferreira JJ, Santos AT, Domingos J, et al. Clinical Parameters and Tools for Home-Based Assessment of Parkinson's Disease: Results from a Delphi study. Journal of Parkinson's disease 2015;5(2):281-290.
